# Amplification of a radially polarised beam in an Yb:YAG thin-slab

**DOI:** 10.1007/s00340-017-6802-z

**Published:** 2017-08-05

**Authors:** C. R. Smith, S. J. Beecher, J. I. Mackenzie, W. A. Clarkson

**Affiliations:** Optoelectronics Research Centre, University of Southampton, Southampton, SO17 1BJ UK

## Abstract

The use of an Yb:YAG thin-slab architecture for amplification of a radially polarised beam at 1030 nm is investigated and shown to be a promising route for power scaling. The detrimental impact of the Gouy phase shift on radial polarisation purity is considered and a simple scheme for effective phase shift management to restore polarisation purity is presented. Preliminary experiments based on a double-pass amplifier configuration yielded an output beam with a high radial polarisation extinction ratio of 15 dB and no degradation in polarisation purity despite the non- axial symmetry of amplifier gain medium. At 50 W of launched pump power a small-signal gain of 7.5 dB was obtained for a 25 mW input, whilst 4.4 dB gain was obtained for a 1.45 W input. The prospects for further power scaling are discussed.

## Introduction

Cylindrical vector (CV) beams have unique characteristics whereby the polarisation state across the beam cross-section is not uniform, as with linear or circularly polarised light, but in fact forms an axially symmetric distribution. Consequently, this leads to an intensity null at the beam centre owing to the polarisation discontinuity. Radially and azimuthally polarised beams form an interesting subset of CV beams, where the polarisation direction is orientated in the radial and tangential directions, respectively. Numerous applications for these exotic beams have been identified including particle acceleration [1] and particle trapping [2], which utilise the intense longitudinal electric field produced by a strongly focused radially polarised beam. A remarkable feature of radially polarised beams is their ability to be focused to smaller spot sizes than linearly polarised beams, leading to applications in high-resolution imaging [3]. Material processing, such as the cutting and drilling of metals, is another area where these beams have attracted great interest. It has been shown that cutting efficiency can be 1.5-2 times higher using radial polarisation rather than circular polarisation [4], and drilling can be 1.5–4 times more efficient with azimuthal polarisation compared to linear and circular polarisation [5].

Various methods of producing CV beams have been implemented over the decades. CV beam generation can be broadly divided into two distinct categories; intracavity and extracavity techniques. Intracavity techniques rely on a cavity design that ensures one polarisation, i.e. radial or azimuthal, is dominant over the other and is, therefore, emitted from the laser directly. One popular approach is to exploit thermally induced bi-focusing in host materials such as YAG to render the resonator unstable for one polarisation, or yield a lower threshold for one polarisation by virtue of having a more favourable spatial overlap with the inversion distribution [6]. Alternatively, intracavity elements to provide polarisation discrimination, such as calcite crystals [7], Brewster-angled axicons [8], conical Brewster prisms [9], grating mirrors [10], or nanograting waveplates [11] can be employed. Extracavity techniques typically convert more traditional polarisation states, such as linear or circular polarisation, into radial or azimuthal polarisation. This can be achieved interferometrically, through the combination of orthogonal linearly polarised HG_01_ and HG_10_ beams [12]. Alternatively, a spatially variable retardation plate can be used to convert linear polarisation to radial or azimuthal polarisation [13].

To realise their potential within material processing and other applications, CV beams must be produced with sufficient power. Therefore, a system that produces CV beams amplified to high powers is desirable. Various architectures have been employed for CV beam amplification, including thin-disc [14], fiber [15, 16], single crystal fiber [17] and rod [18]. It is critically important to avoid depolarisation effects during amplification. This is achieved in the thin-disc configuration by geometry, which minimises thermally induced bifocusing with a predominantly one-dimensional heat-flow. The axial symmetry of the other architectures leads to different focal lengths for radial and azimuthal polarised beams when thermally stressed. Furthermore, a pure radial/azimuthal polarisation state will not experience depolarisation loss. However, to the best of our knowledge, there are no reports of amplification of CV beams in slab architectures, which lack axial symmetry. An important advantage of slab designs over their rod counterparts is a larger cooling surface area in close proximity to the pumped region, which allows effective one-dimensional heat removal and, as a consequence, reduced thermal lensing and an increased thermal fracture limit. Additionally, amplification in a slab is simpler than in a thin-disc, which due to the very short interaction length requires a complex multi-pass arrangement to achieve an appreciable gain. These benefits have allowed slab amplifiers to produce 400 W of nearly diffraction limited output power [19], and 1.1 kW of multimode output power [20].

In this paper, we demonstrate that the polarisation purity of CV beams can be maintained after amplification in a thin-slab, opening up a new route to power scaling. We begin in Sect. 2 by describing the Yb:YAG seed laser, which exploits thermally induced bi-focussing for direct generation of a radially polarised beam with good beam quality and high polarisation purity. Section 3 describes the Yb:YAG thin-slab amplifier used to amplify the seed beam, taking into account the important consequences of the Gouy phase shift, and how this must be managed to retain radial polarisation purity. In Sect. 4 preliminary results will be presented, confirming the validity of this approach. Finally, in Sect. 5, we discuss the scope for further optimisation and the prospects for improved performance along with the main conclusions.

## Radially polarised Yb:YAG seed source

**Fig. 1:**
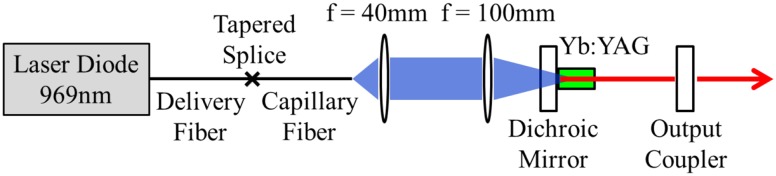
Schematic of the radially polarised seed source

**Fig. 2:**
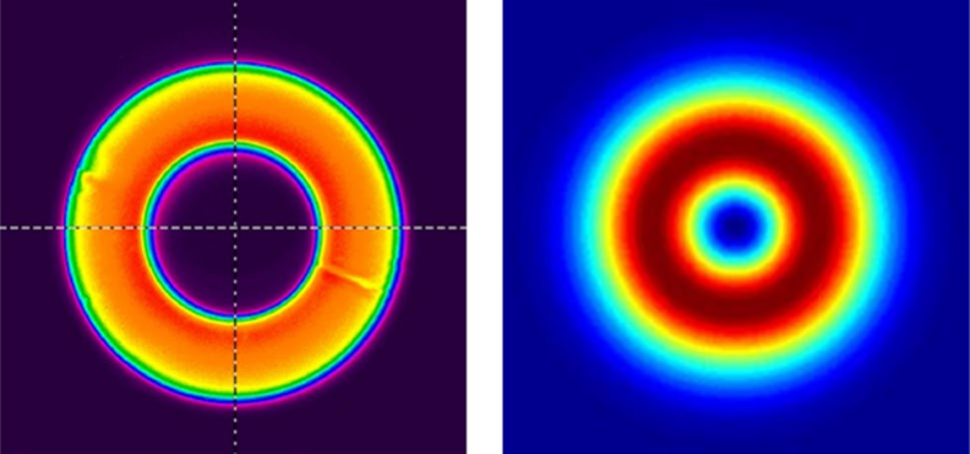
Measured pump intensity profile and theoretical intensity profile of the LG_01_ mode

The Yb:YAG seed laser used in our experiments (see Fig. 1) employed a simple two-mirror cavity design comprising a plane pump incoupling mirror with high reflectivity (*R* > 99.8%) at the lasing wavelength (1030 nm) and high transmission (*T* > 95%) at the pump wavelength (969 nm) and a plane output coupler with 5% transmission at 1030 nm. A 3-mm long, 2-mm diameter Yb:YAG rod was employed as the gain medium. The latter had antireflection coated faces at both pump and lasing wavelengths, and was mounted in a water-cooled copper heat-sink at a distance of approximately 2 mm from the pump incoupling mirror. Pump light was provided by a fibre-coupled laser diode at 969 nm. The pump delivery fibre had core and cladding diameters of 105 and 125 μm, respectively. The output from this fibre was re-shaped to yield an annular near-field beam profile with the aid of a capillary fibre with an inner-hole diameter of 100 μm and an outer pump guide diameter of 200 μm. The end section of this fibre was tapered to a solid-glass structure of 125 μm diameter to allow splicing to the pump delivery fibre with <1% transmission loss. The output beam from the capillary fibre was relay-imaged using a simple telescope with a magnification factor of 2.5 to yield a near-field annular pump profile (see Fig. 2) and hence inversion profile in the Yb:YAG rod. This provides a good spatial overlap with the desired radially polarised LG_01_ mode, with the ideal theoretical profile shown in Fig. 2.

Preferential lasing on the doughnut-shaped radially polarised lasing mode was achieved by carefully adjusting the cavity length (under pumping) exploiting bi-focusing to achieve a more favourable spatial overlap with the pumped region and hence a lower threshold for the radially polarised LG_01_^*^ mode compared to the azimuthally polarised LG_01_ mode.

**Fig. 3:**
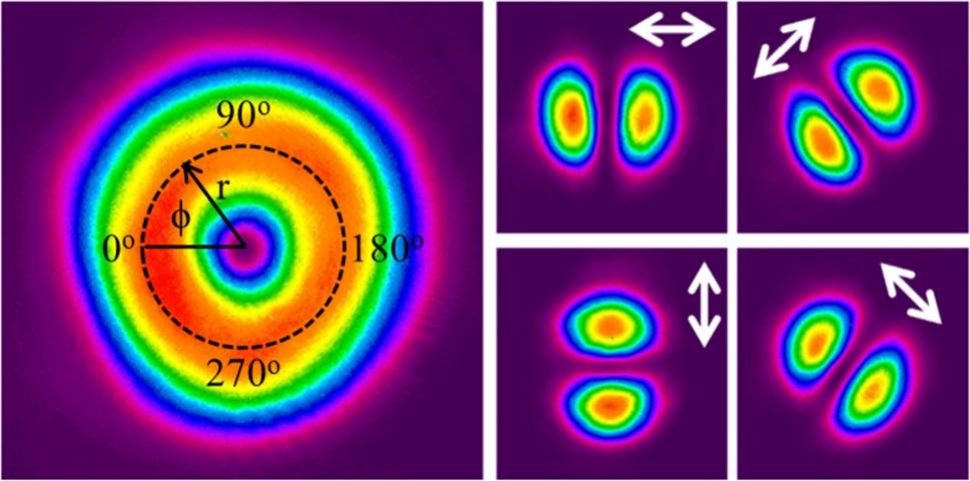
Intensity profile of the generated radially polarised LG_01_^*^ mode (*left*) and intensity profiles after the beam has passed through a polariser orientated at the angles indicated by the *arrows*

For a cavity length of 57 mm 1.45 W of radially polarised output was produced with a corresponding optical efficiency of ~38%. The intensity profile, shown in Fig. 3, confirms that a high quality doughnut beam was produced. The beam propagation (*M* ^2^) factor was measured utilising the D4σ method, using a CCD camera to measure the beam sizes of a focused beam over an appropriate range. A value of 2.3 was obtained for the beam propagation factor, which is in close agreement with the theoretical value of 2. Also shown in Fig. 3 are the intensity profiles of the beam after passing through a linear polariser orientated at the angles depicted by the arrows. The clean two-lobed structure indicates excellent radial polarisation purity.

A quantitative value for the radial polarisation extinction ratio (RPER) was determined via the following approach [11]: The intensity of the two-lobed profiles after transmission through a linear polariser were measured along a circular path of radius *r* from the beam centre, where r is selected to maximise the intensity. A given point on the circle can be described by an azimuthal angle, *ϕ*, ranging from 0°–360°, as shown in Fig. 3. The angle of the polariser is defined by *ϕ*_p_. For a perfect radially polarised beam one would expect the intensity at each radial and azimuthal position, *I(r,ϕ)*, to adhere to the following relation:

1 $$ I\left( {r,\varphi } \right) = I\left( r \right)\cos^{2} \left( {\phi - \phi_{\text{p}} } \right). $$

**Fig. 4:**
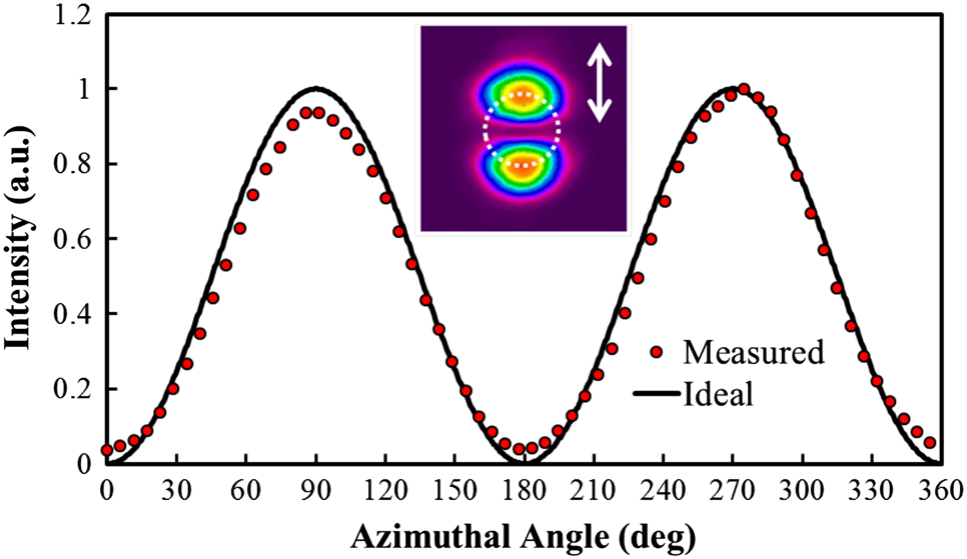
Measured and ideal intensity profile as a function of azimuthal angle for the radially polarised beam after it has passed through a linear polariser orientated in the direction of the *arrow*

The measured data and the modelled intensity for a beam with perfect polarisation purity are shown in Fig. 4 for a single polariser orientation at *ϕ*_p_ = 90°. To determine the RPER, the average maximum intensity at *ϕ* = 90° and *ϕ* = 270° is divided by the average minimum intensity at *ϕ* = 0° and *ϕ* = 180° to yield an extinction ratio. The final RPER is the average of this extinction ratio over four arbitrarily chosen polariser orientations: *ϕ*_p_ = 0°, 90°, 180° and 270°. The RPER of the source was > 15 dB, confirming high radial polarisation purity.

## Amplifier configuration

**Fig. 5:**
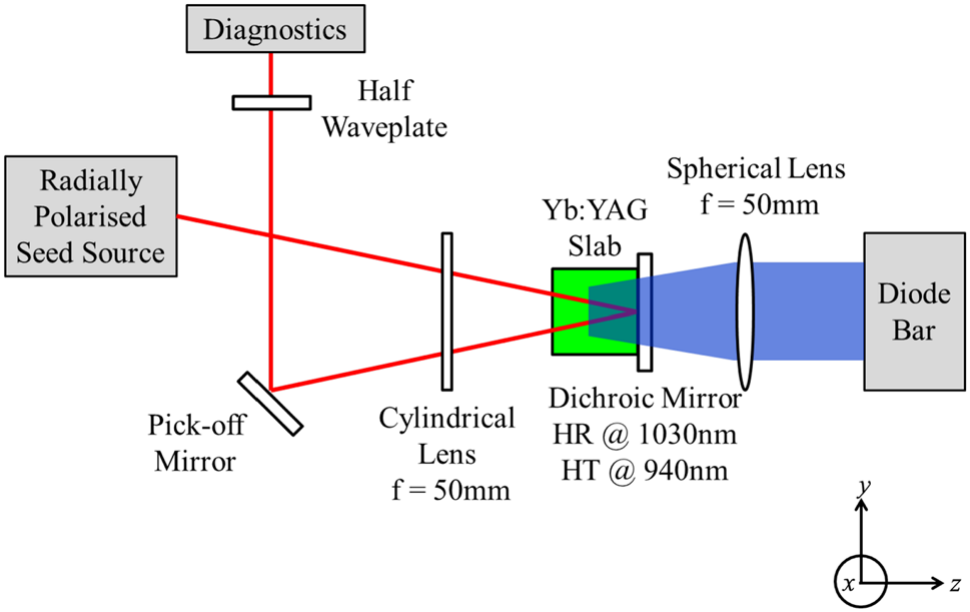
Yb:YAG slab amplifier arrangement

The set-up used for amplification of the radially polarised seed beam is shown in Fig. 5. The gain medium was an Yb:YAG slab with transverse dimensions 1 mm × 5 mm by 5 mm long and 5 at.% Yb^3+^ doping concentration. The slab was mounted in a copper heat-sink arranged to provide conduction cooling of the 5 mm × 5 mm faces, and hence heat flows predominantly in the *x* direction. The end faces were antireflection coated at the pump and seed wavelengths. Pump light at 940 nm was provided by a 50 W diode-bar collimated in the fast direction (*x*) by a cylindrical microlens and in the slow direction (*y*) by a cylindrical microlens array. The resulting beam was focussed into the slab with the aid of an antireflection coated spherical lens of focal length 50 mm to yield a rectangular inversion region with approximate waist transverse dimensions of 85 μm (*x* direction) × 1 mm (*y* direction), and hence an aspect ratio of ~12:1. Over 90% of the pump radiation was absorbed in a single-pass. The radially polarised seed source was directed into the Yb:YAG at a small angle of incidence (~3°) whilst being focused in the *x* direction with an antireflection coated cylindrical lens of focal length 50 mm. This arrangement yielded a seed beam profile that was approximately spatially matched to the inversion distribution, with beam dimensions of ~55 μm (*x* direction) and ~700 μm (*y* direction) in the slab. After a first pass through the slab the seed beam was reflected by a plane dichroic mirror with high reflectivity (>99.8%) at 1030 nm and high transmission at 940 nm to pass through the slab for a second time with the exiting beam re-collimated in the *x* direction by the cylindrical lens before being re-directed to the beam diagnostics.

**Fig. 6:**
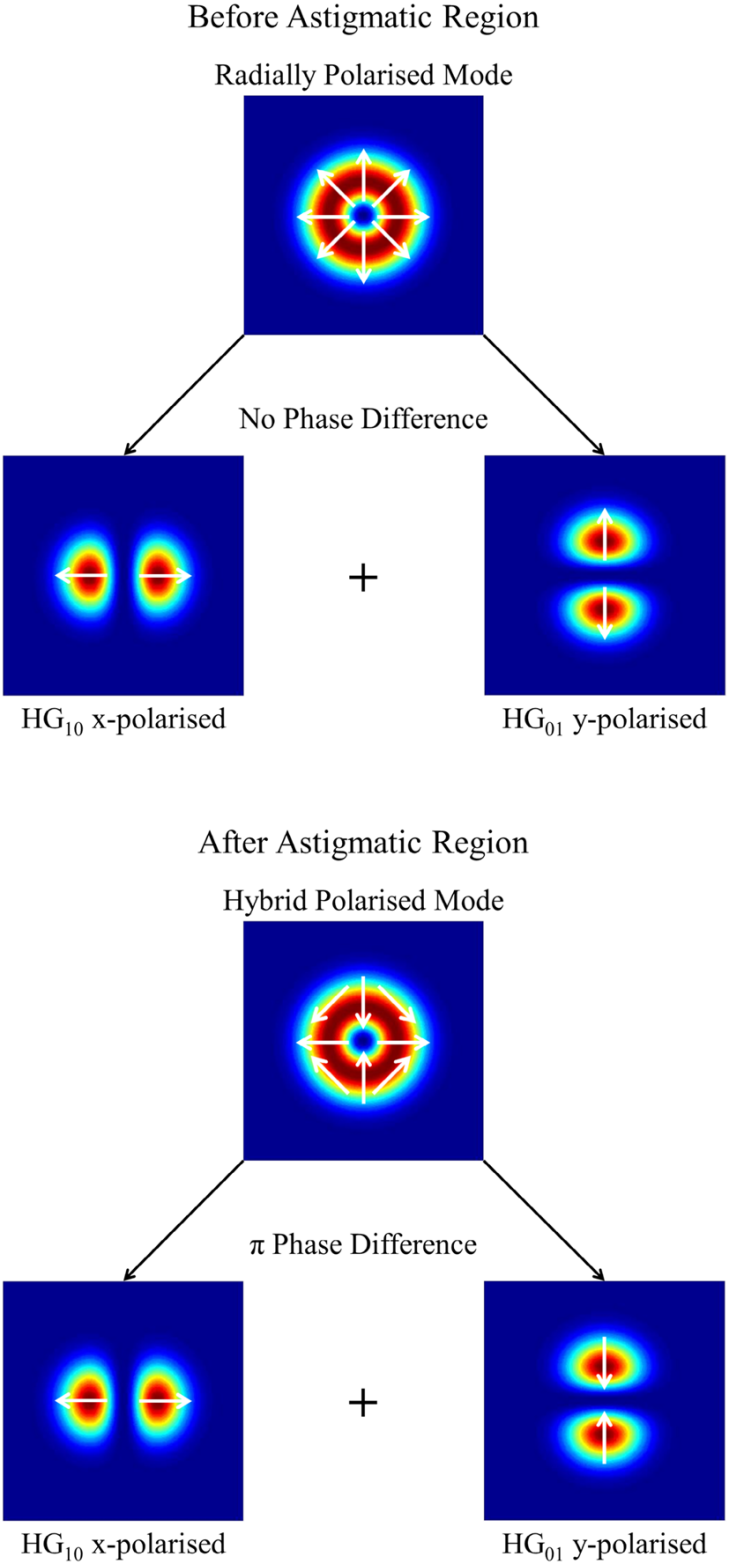
Computation of radially polarised input (*top*) and hybrid output mode (*bottom*)

The amplification set-up involves astigmatic focusing and subsequent re-collimation of the amplified beam with the cylindrical lens. This has a very detrimental impact on the polarisation purity due to the different Gouy phase shifts of the constituent Hermite-Gaussian (HG) modes. The radially polarised LG_01_ seed beam can be considered as a coherent superposition of a HG_10_ mode polarised in the *x* direction and a HG_01_ mode polarised in the *y* direction, as shown in the theoretical computation in Fig. 6 (top). In general, the Gouy phase, *Ψ*_G_, for each HG_nm_ component in the astigmatic region evolves as [21]:

2 $$ \varPsi_{\text{G}} = 2\left[ {\left( {n + \frac{1}{2}} \right){ \arctan }\left( {\frac{2f}{{z_{\text{Rx}} }}} \right) + \left( {m + \frac{1}{2}} \right){ \arctan }\left( {\frac{2f}{{z_{\text{Ry}} }}} \right)} \right], $$

where *2f* is the effective path length between the focusing and re-collimating cylindrical lens for the beam in the *x* direction (i.e. twice the focal length), and *z*_Rx_ and *z*_Ry_ are the corresponding Rayleigh ranges in the *x* and *y* directions, respectively. This assumes that the beam is non-astigmatic in the regions before focusing and after re-collimation, which is the case in this configuration. Therefore, through the astigmatic region the phase of each HG_nm_ mode evolves differently. Once the beam has been re-collimated and enters the non-astigmatic region, the Gouy phase of each HG_nm_ mode will evolve identically, thus any phase difference that occurred during passage through the astigmatic region will be maintained. As the beam is focused in the *x* direction, *z*_Rx_ is significantly smaller than *z*_Ry_ in the astigmatic region. Consequently, in this set-up, it can be assumed that *2f* >> *z*_Rx_ and *2f* << *z*_Ry_ (2*f* = 100 mm, *z*_Rx_ ≈ 4 mm, *z*_Ry_ ≈ 650 mm). Hence, in Eq. (2), the inverse tangent of the first term tends to *π*/2 whilst the inverse tangent of the second term tends to 0. One can subsequently deduce that the Gouy phase of the HG_10_ mode will evolve by 3*π*/2, whilst the Gouy phase of the HG_01_ mode will evolve by *π*/2 in the astigmatic region. Consequently, a net *π* phase-shift is established between the orthogonally polarised HG_10_ and HG_01_ modes. The amplified output beam (i.e. after re-collimation) is the superposition of these modes and the resultant polarisation is a hybrid radial-azimuthal polarisation distribution [see Fig. 6 (bottom)]. To recover a pure radially polarised beam a half-waveplate with its fast axis orientated in the *x* or *y* direction is incorporated into the output arm of the amplifier configuration after re-collimation. This creates a further *π* phase-shift between the HG_10_ and HG_01_ modes, hence compensating for the Gouy phase shift introduced during passage through the astigmatic region, yielding a radially polarised output beam.

## Results for amplifier performance

**Fig. 7:**
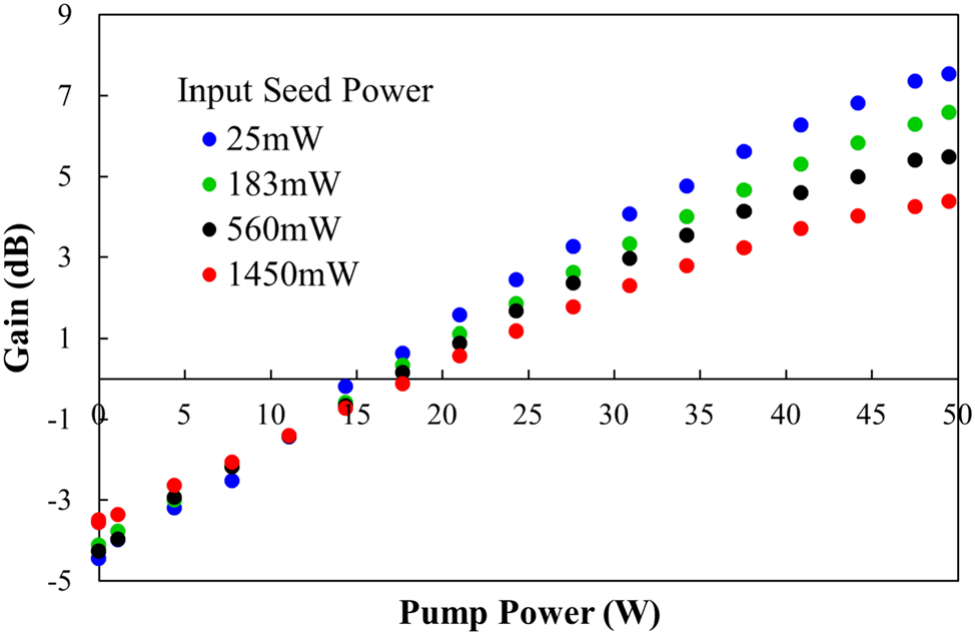
Measured amplifier gain for various seed powers as a function of pump power

**Fig. 8:**
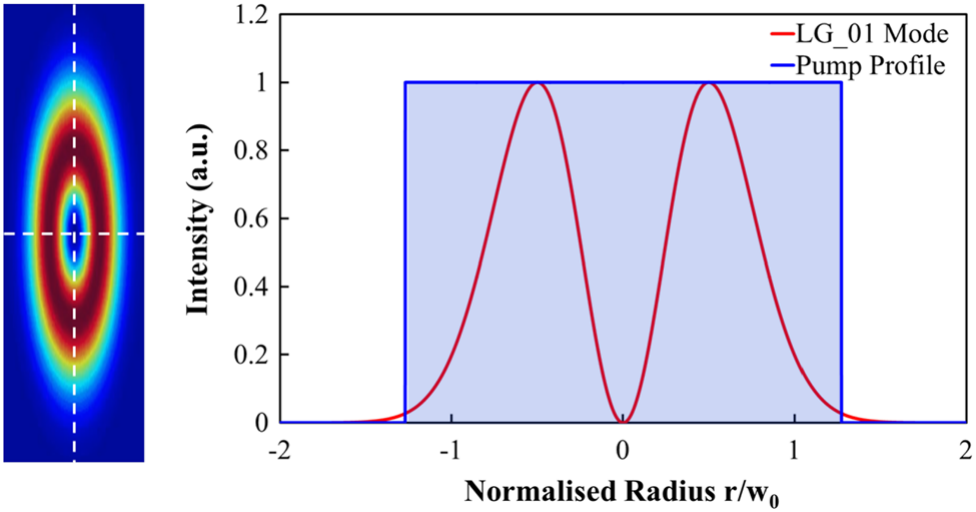
Schematic showing the computed overlap for the LG_01_ mode and pump profile in the slab. Overlaid beam profiles shown in 2D (*left*) and through beam centre (*right*)

Figure 7 shows the measured gain as a function of pump power for the Yb:YAG slab arrangement shown in Fig. 5. Amplifier gain was measured for a range of input seed powers which were varied external to the Yb:YAG seed laser with the aid of appropriate partially reflective dielectric mirrors to preserve the seed beam characteristics. As the seed power launched into the amplifier increases the gain decreases, as expected due to gain saturation. Note that internal losses of the system are not accounted for in the gain values. For 25 mW of input seed power a gain of 7.5 dB was achieved at the maximum diode pump power of 50 W, which decreases to 4.4 dB for an input seed power of 1.45 W. This corresponds to a maximum amplified output power of ~4 W, with an extraction efficiency of 5.7%. The amplifier extraction efficiency was limited by the available pump power and by non-optimum spatial overlap of the inversion region with the seed beam profile. The seed beam cannot extract inversion in the intensity null central region of the doughnut profile. This is shown schematically in Fig. 8, where we depict the beam overlap in the slab for an LG_01_ mode with radius w_0_ and a top-hat pump beam. Figure 8 (left) shows a 2D representation of the overlap, whilst Fig. 8 (right) shows the beam intensity profiles through the centre of the beams (white dashed lines). Therefore, to improve the extraction efficiency, pump shaping techniques should be employed to minimise the pump intensity in the central region of the seed beam.

**Fig. 9:**
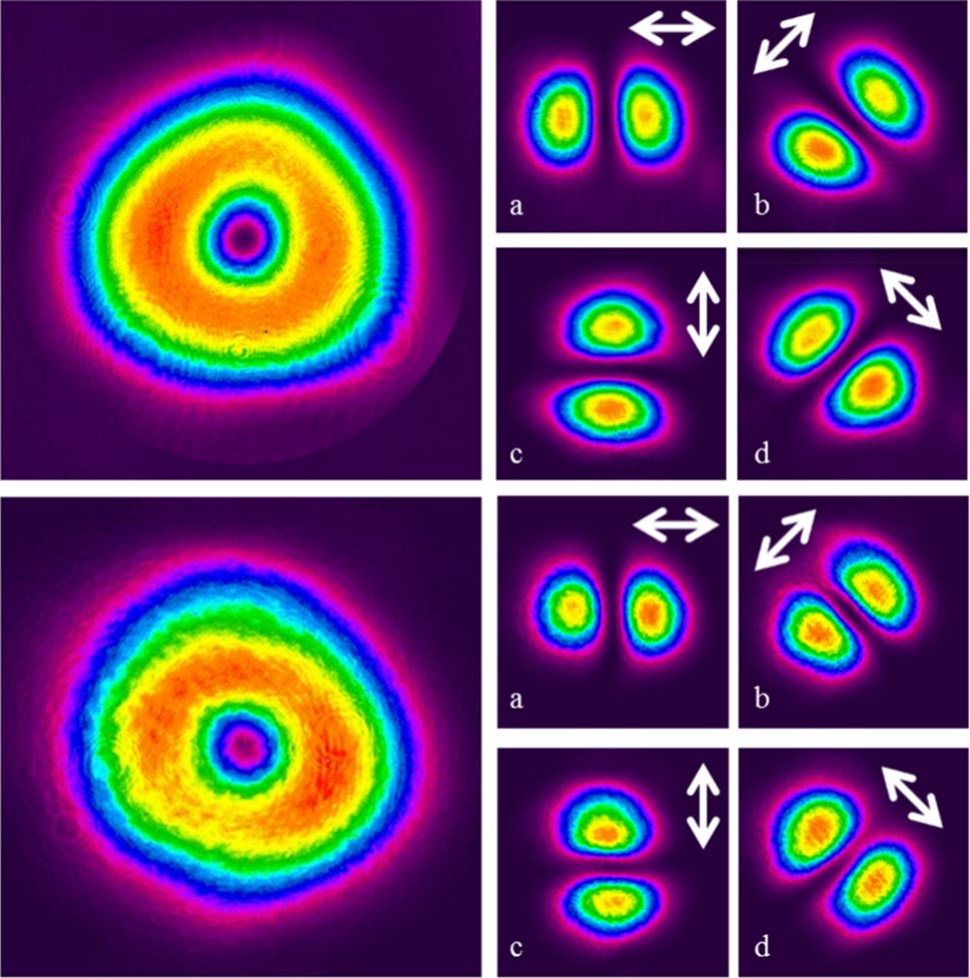
Intensity profile of the output beam at maximum gain level and intensity profiles after the beam has passed through a polariser orientated at the angles indicated by the arrows for the 25 mW input beam (*top*) and 1.45 W input beam (*bottom*)

**Fig. 10:**
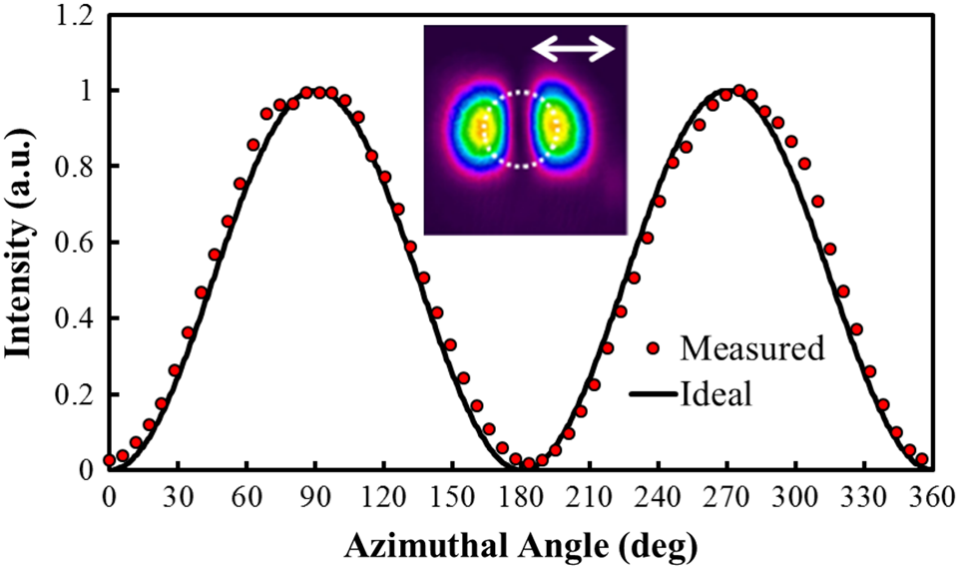
Measured and ideal intensity profile as a function of azimuthal angle for the amplified radially polarised beam after it has passed through a linear polariser orientated in the direction of the *arrow*

Crucially, the beam profiles and polarisation purity were maintained for the amplified output beam over the full range of pump and seed powers available, despite the highly asymmetric nature of the gain region and associated thermal effects. Figure 9 shows the intensity profile of the amplified beam at the maximum gain level for the lowest and highest seed powers, along with the corresponding intensity profiles after the beam had passed through a linear polariser orientated at the angles depicted by the white arrows. One can clearly see that the beam intensity profile is preserved along with the high degree of radial polarisation purity. It is worth noting that without the half-waveplate providing the necessary compensation, the computed hybrid mode in Fig. 6 was produced. In terms of the beam intensity profile, the horizontal and vertical lobes (i.e. a and c in Fig. 9) remained unchanged, whilst the diagonal profiles (i.e. b and d) were exchanged. Beam quality measurements show that the beam propagation factor degraded only very slightly to *M* ^2^ = 2.3 for the lowest seed input power, and *M* ^2^ = 2.4 for the highest seed input power. Figure 10 shows an example of a RPER plot for a single polariser orientation at the maximum gain level for the lowest seed input power. The measured intensity agrees well with the ideal scenario, and the RPER was maintained >15 dB for the lowest and the highest seed input powers.

## Conclusion

In this paper, we have demonstrated that radially polarised beams, a subset of CV beams, can be amplified in a highly asymmetric slab gain medium whilst maintaining beam quality and, most importantly, radial polarisation purity. The requirement for astigmatic focusing of the seed laser beam to match the rectangular inversion region leads to change in the polarisation that results from different Gouy phase shifts of the constituent orthogonally polarised HG_10_ and HG_01_ beams. We have shown that this can be compensated in a very simple, power-scalable manner by the inclusion of a half-wave plate in the output beam path. Preliminary amplifier experiments yielded a gain of 7.5 dB for a 25 mW seed input power and 4.4 dB for a 1.45 W input power. The beam propagation factor was only slightly degraded to *M* ^2^ = 2.3 and *M* ^2^ = 2.4 at maximum gain for the lowest and highest seed input powers, respectively, whilst the RPER was maintained at >15 dB for both cases. The overall performance of the amplifier was limited by non-optimum spatial overlap of the inversion profile with the seed beam profile and by available pump power. Noting the power scaling potential of slab gain media, we anticipate that the availability of higher pump power along with further optimisation of the pump beam profile should offer a pathway to considerably higher powers. It is also worth pointing out that this approach is equally applicable to azimuthally polarised beams and hence will be of interest for a range of applications (and particularly laser processing of materials), where high power CV beams can yield significant benefits.

## Supplementary material

The data from this paper can be obtained from University of Southampton e-Prints repository Dataset for Amplification of radially polarised beam in an Yb:YAG thin-slab (http://doi.org/10.5258/SOTON/D0181).
